# Bone Marrow Mesenchymal Stem Cells Expanded Inside the Nichoid Micro-Scaffold: a Focus on Anti-Inflammatory Response

**DOI:** 10.1007/s40883-023-00296-z

**Published:** 2023-03-20

**Authors:** Bianca Barzaghini, Stephana Carelli, Letizia Messa, Federica Rey, Maria Antonietta Avanzini, Emanuela Jacchetti, Erika Maghraby, Clarissa Berardo, Gianvincenzo Zuccotti, Manuela Teresa Raimondi, Cristina Cereda, Valeria Calcaterra, Gloria Pelizzo

**Affiliations:** 1 Department of Chemistry, Materials, and Chemical Engineering “Giulio Natta,” Politecnico Di Milano, Milan, Italy; 2 Pediatric Research Center “Romeo Ed Enrica Invernizzi,” Department of Biomedical and Clinical Sciences, University of Milan, Milan, Italy; 3 Center of Functional Genomics and Rare Diseases, Department of Pediatrics, Buzzi Children’s Hospital, Milan, Italy; 4 Department of Electronic, Information and Bioengineering, Politecnico di Milano, Milan, Italy; 5 Immunology and Transplantation Laboratory, Cell Factory, Pediatric Hematology Oncology, Fondazione IRCCS Policlinico S. Matteo, Pavia, Italy; 6 Department of Biology and Biotechnology “L. Spallanzani”, University of Pavia, Pavia, Italy; 7 Department of Pediatrics, Buzzi Children’s Hospital, Milan, Italy; 8 Department of Internal Medicine, University of Pavia, Pavia, Italy; 9 Pediatric Surgery Unit, Buzzi Children’s Hospital, Milan, Italy; 10 Department of Biomedical and Clinical Science, University of Milan, Milan, Italy

**Keywords:** Regenerative medicine, Mesenchymal stem cells, Nichoid scaffold, Engineered stem cell niche

## Abstract

**Purpose:**

Mesenchymal stem cells (MSCs) represent a promising source for stem cell therapies in numerous diseases, including pediatric respiratory system diseases. Characterized by low immunogenicity, high anti-inflammatory, and immunoregulatory features, MSCs demonstrated an excellent therapeutic profile in numerous in vitro and preclinical models. MSCs reside in a specialized physiologic microenvironment, characterized by a unique combination of biophysical, biochemical, and cellular properties. The exploitation of the 3D micro-scaffold Nichoid, which simulates the native niche, enhanced the anti-inflammatory potential of stem cells through mechanical stimulation only, overcoming the limitation of biochemical and xenogenic growth factors application.

**Materials and Methods:**

In this work, we expanded pediatric bone marrow MSCs (BM-MSCs) inside the Nichoid and performed a complete cellular characterization with different approaches including viability assays, immunofluorescence analyses, RNA sequencing, and gene expression analysis.

**Results:**

We demonstrated that BM-MSCs inside the scaffold remain in a stem cell quiescent state mimicking the condition of the in vivo environment. Moreover, the gene expression profile of these cells shows a significant up-regulation of genes involved in immune response when compared with the flat control.

**Conclusion:**

The significant changes in the expression profile of anti-inflammatory genes could potentiate the therapeutic effect of BM-MSCs, encouraging the possible clinical translation for the treatment of pediatric congenital and acquired pulmonary disorders, including post-COVID lung manifestations.

**Lay Summary:**

Regenerative medicine is the research field integrating medicine, biology, and biomedical engineering. In this context, stem cells, which are a fundamental cell source able to regenerate tissues and restore damage in the body, are the key component for a regenerative therapeutic approach. When expanded outside the body, stem cells tend to differentiate spontaneously and lose regenerative potential due to external stimuli. For this reason, we exploit the scaffold named Nichoid, which mimics the in vivo cell niche architecture. In this scaffold, mesenchymal stem cells “feel at home” due to the three-dimensional mechanical stimuli, and our findings could be considered as an innovative culture system for the in vitro expansion of stem cells for clinical translation.

**Future Perspective:**

The increasing demand of safe and effective cell therapies projects our findings toward the possibility of improving cell therapies based on the use of BM-MSCs, particularly for their clinical translation in lung diseases.

**Graphical Abstract:**

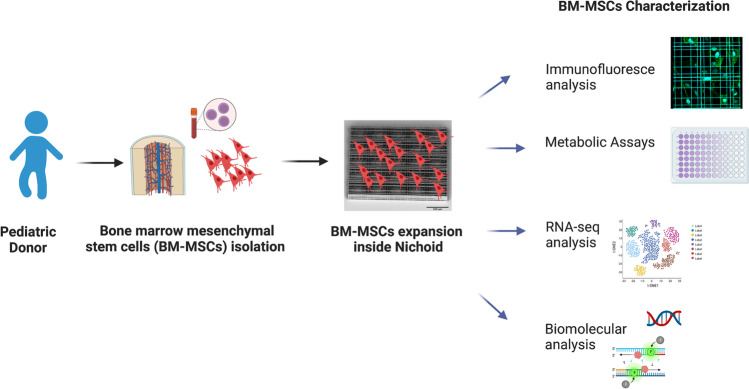

## Introduction

A regenerative approach based on stem cell potential is nowadays studied as a potential treatment for respiratory system diseases in adult as well as pediatric patients [1, 2]. To this end, the use of mesenchymal stem cells (MSCs) could be of crucial interest as these are primary candidates for cellular therapies and tissue engineering [3]. Indeed, MSCs are multipotent cells that can self-renew and differentiate into multiple tissue-forming cell lineages [4], showing secretion [5], homing [6], and immunomodulatory properties.

For these reasons, MSCs were found to have a good therapeutic efficacy in congenital and acquired pulmonary diseases, including SARS-COV-2 infection [7, 8], as demonstrated in pre-clinical studies and clinical trials performed in both adult and pediatric patients [9–11]. Indeed, both congenital interstitial lung disease [12, 13] and pulmonary injury due to infectious etiology (SARS, H1N1, COVID-19) appear to be characterized by inflammatory and fibrotic changes in the lung parenchyma, leading to gas exchange impairment and chronic respiratory failure associated with high morbidity and mortality. In this setting, MSCs regulate immune and inflammatory responses, thus supporting reparative and regenerative processes. Nowadays, 285 MSCs clinical trials involve pediatric patients affected by different diseases, including osteogenesis imperfecta, perinatal arterial ischemic stroke, diabetes, and pneumonia. Unfortunately, only 10 registered clinical trials were completed or reached the recruitment for phase III. The lack of therapeutic effect could be related to the conventional systems for stem cell expansion, which do not mimic the physiological environment, causing spontaneous stem cell differentiation and senescence [14, 15]. Several research groups overcame this limitation using biochemical or exogenous growth factors, such as leukemia inhibitor factor (LIF) and bovine serum albumin (BSA), for basic research [16, 17]. From a regulatory point of view when referring to clinical translation, the addition of exogenous factors represents a cellular manipulation, and this is considered a risk when the cells or their products must be transferred to pediatric patients [18, 19]. In this context, the use of 3D scaffolds or biomaterials to better simulate a physiological ex-vivo environment is becoming more relevant. Indeed, in 2019, Chen et al. studied a 3D-printed cartilage extracellular matrix (ECM)/gelatin methacrylate (GelMA) loaded with MSCs exosomes for the treatment of osteoarthritis [20]. The following year, Ji et al. demonstrated a possible application of chitin-derived hydrogels to encapsulate MSCs for bone regeneration [21]. Finally, a recent publication by Dong et al. exploited an electrospun polycaprolactone/silk fibroin scaffold functionalized with MSC-derived extracellular matrix [22].

Alternative mechanical-induced culture methods have been proposed to maintain or even increase the therapeutic properties of stem cells, and among them, there is the micro-engineered three-dimensional substrate Nichoid, which recapitulates the architecture of the physiological niche in vitro [23]. This scaffold mimics the physiological environment of MSCs, and thus, the stemness features and the immunomodulatory properties of these cells are maintained or even increased. Indeed, previous studies demonstrated the Nichoid’s ability to affect stem cells’ migration and proliferation [23–29]. This approach was also found to enhance the pluripotency and differentiation capabilities of stem cells isolated from different sources simply through the arrangement of geometric and physical distribution [30–32]. Recent studies also confirmed the enhanced therapeutic efficacy of Nichoid-cultured neural precursors cells in an animal model of Parkinson’s disease [30] and the molecular signature associated with mechanotransduction processes induced simply through the Nichoid mechanical stimulation [31, 32]. It is for this reason that the expansion of MSCs inside the Nichoid scaffold could represent a potential therapeutic prospect for respiratory diseases. For the first time, in this study, we performed a comprehensive characterization of pediatric bone marrow MSCs (BM-MSCs) expanded inside the Nichoid in terms of viability, morphology, and gene expression analysis, opening the possibility to the exploitation of this micro-scaffold as a stem cell expansion platform for translational medicine, including pediatric lung disease.

## Materials and Methods

### Mesenchymal Stem Cells

BM-MSCs were isolated and expanded from residual bone marrow cells harvested from a female pediatric 10 years old patient, after signing an informed consent, as previously reported in [33], that have been used in hematopoietic stem cell transplantation (HSCT) [33]. Mononuclear cells were isolated by density gradient separation and plated at the density of 160.000 cells/cm^2^ in complete medium (Dulbecco’s modified Eagle’s medium (D‐MEM-Low Glucose + Glutamax) (Gibco) supplemented with 5% human platelet lysate (Macopharma), and 1% Gentamicine (Gibco)). Cultures were kept at 37 °C and 5% CO_2_ in a humidified atmosphere. After 48 h, non-adherent cells were removed, and culture medium was replaced twice a week. At confluence, ≥ 80% MSCs were detached with recombinant Trypsin–EDTA (Euroclone) and replated. BM-MSC surface antigens were evaluated by flow cytometry using CD73, CD90, CD105, and class I-HLA as positive markers and CD34, CD14, CD45, and CD31 (all antibodies from Beckman Coulter) as negative markers. Briefly, 1 × 10^5^ cells/tube were incubated at 4 °C with FITC or PE-conjugated monoclonal antibodies. Ten thousand events were acquired by the FACS Navios flow cytometer (Beckman Coulter). Analysis was performed by Navios software (Beckman Coulter).

### Microfabrication of Nichoids

3D micro-scaffold Nichoids were fabricated on circular glass coverslips 150–170 μm thick and with a 12 mm diameter (BioOptika) previously covered with 23 μl of SZ2080 photoresist by drop-casting. Nichoid microfabrication through two-photon polymerization was done as previously reported [34]. Samples were developed in solvent solution to remove the unpolymerized resin and characterized through scanning electron microscopy (Phenom Pro, Phenom World) to ensure their integrity. Before cell culture, Nichoids were sterilized by washing with deionized water, then they were covered with ethanol 70% for 1.5 h and then exposed to UV radiation for 1 h.

### Cell Seeding Inside the Nichoid Scaffold

1 × 10^4^ BM-MSCs at passage 3–5 were seeded inside the Nichoid and in standard 2D condition (plastic multiwell polystyrene) in a single drop of 35 μl of culture medium. The multi-well was then kept in the incubator for a minimum of 1 h to allow cells to enter inside the niches. After that, the Nichoid was covered with complete medium.

### Immunocytochemistry Analysis

Cells were seeded inside the Nichoid and on ethanol-washed glass coverslips as 2D control. After 7 days of cell expansion, cells were fixed with 4% paraformaldehyde in 0.1 M PBS (Thermo Fisher Scientific), pH 7.4, for 20 min at room temperature, and then washed with PBS following the protocol reported in [31, 35]. The coverslips were incubated overnight at 4 °C in PBS containing 10% normal goat serum (NGS, Thermo Fisher Scientific), 0.3% Triton X-100 (BDH, VWR), and the appropriate primary antibody. Cell features were assessed with antibodies against Vimentin (1:200 Immunological Sciences). To visualize alpha-actin filaments, cells were stained using Phalloidin, Fluorescein Isothiocyanate Labeled (Phalloidin-FITC, 1:50, Sigma). Nuclei were stained with Hoechst 33342 (Invitrogen). Then, BM-MSCs were rinsed with PBS and 10% NGS and incubated with the appropriate secondary antibody (Alexa Fluor® 488 and 647, Thermo Fisher Scientific) for 1.5 h. Samples were mounted using Prolong glass antifade Mountant (Invitrogen, Thermo Fisher Scientific) and analyzed by confocal microscopy (Olympus FluoView FV10i). As a control, the appropriate secondary antibody was administrated omitting the primary one (Alexa Fluor® 488 or 647, Thermo Fisher Scientific). Immunocytochemistry analysis was performed acquiring three different fields for each sample for three independent experiments.

### Alamar Blue Assay

The Alamar Blue assay (Thermo Fisher) is a metabolic assay that uses the reducing power of living cells as a cellular health indicator [36]. Following the manufacturer’s instructions, from a stock solution of 0.2 mg/ml of resazurin solution, a dilution of 1:10 was used for each sample, and the multiwell was incubated for 24 h in the dark and avoiding light exposure inside the incubator. The solution was then transferred in a 96 multiwell plate, and the absorbance at 570 nm and 600 nm was detected with a Multiskan GO spectrophotometer (Thermo Fisher Scientific).

The equation applied to determine the percent reduction of resazurin is the following:

$$\%\mathrm{Reduced}=\frac{C_{\mathrm{red}}\mathrm{Test}}{C_{\mathrm{ox}}\mathrm{NegativeControl}}=\frac{{(\varepsilon}_{\mathrm{ox}})\lambda_2A\lambda_1-{(\varepsilon}_{\mathrm{ox}})\lambda_1A\lambda_2}{{(\varepsilon}_{\mathrm{RED}})\lambda_1A'\lambda_2-{(\varepsilon}_{\mathrm{RED}})\lambda_2A'\lambda_1}$$

where

*λ*1: 570nm (reduced)
*λ*2: 600nm (oxidized)
*C*_RED_: concentration of reduced form of resazurin
*C*_OX_: oxidized form of resazurin
*ε*_OX_: molar extinction coefficient of resazurin oxidized form
*ε*_RED_: molar extinction coefficient of resazurin reduced form
*A*: absorbance of test wells
*A’*: absorbance of negative control well. The negative control well should be a mix of clean culture media and resazurin.

**Table:** 

Wavelength (*λ*)	*ε*_OX_	*ε*_RED_
570 nm	80,586	155,677
600 nm	117,216	14,652

### RNA Extraction and Library Preparation for RNA-Seq and Bioinformatic Data Analysis

Total RNA from cells expanded for 7 days inside the Nichoid and in control condition was isolated using TRIzol Reagent (Invitrogen) following standard protocol and then quantified using the Multiskan GO spectrophotometer (Thermo Fisher Scientific). Extracted RNA was used for both gene profiling study and PCR analysis. RNA-seq libraries were prepared with the CORALL Total RNA-Seq Library Prep Kit (Lexogen, Vienna, Austria) using 500 ng total RNAs of BM-MSCs-Nichoid and BM-MSCs grown in control condition. The RiboCop rRNA Depletion Kit (Lexogen, Vienna, Austria) was used to remove rRNA. Qualities of sequencing libraries were assessed with D1000 ScreenTape Assay using the 4200 TapeStation System (Agilent) and quantified with Qubit™ dsDNA HS Assay Kit (Invitrogen). BlueBee® Genomics Platform (Lexogen, Austria) pipeline was used to obtain transcript abundance, and the quality of individual sequences was evaluated using FastQC software (version 0.11.9). Adapters were trimmed with Cutadapt software, whereas UMI were removed with UMI tools. Reads were mapped using STAR software (version 2.7) with Gencode Release 27 (GRCh38) as a reference, and transcript intensities were computed using the Mix^2^ RNA-Seq Data Analysis Software (Lexogen, Austria) with the “-strandness forward” option. Differential expression analysis was performed using R package DESeq2, and genes were considered differentially expressed with |log_2_(Nichoid samples/Control samples)|≥ 1 and a False Discovery Rate ≤ 0.1 [37]. Moreover, R software was used to generate heatmaps (heatmap.2 function from the R ggplots package) and PCA plots (prcomp function from the R ggplots package [38]). The NDEx plugin [39] was used to perform analyses concerning the interaction of the differentially expressed genes [40]. The pathways analysis was performed through g:Profiler ordering differentially expressed coding genes according to |log_2_(Nichoid/Control)| and considering the Reactome database [41, 42].

### Real-Time PCR Analysis

A total of 500 ng of RNA were retrotranscribed using the iScript™ Reverse Transcription Supermix for RT-qPCR (Bio-Rad) kit following the manufacturer’s instructions. Real-time PCR was performed with the CFX Connect Real-Time PCR System (Bio-Rad) using SsoAdvanced™ Universal SYBR® Green Supermix (Bio-Rad). The NCBI’s Primer-BLAST (https://www.ncbi.nlm.nih.gov/tools/primer-blast/) tool was used to design primers. Gene expression was calculated using the 2^−ΔΔCt^ method as reported by [43]. GAPDH was used as endogenous control.

Primers used for BM-MSCs analysis:

**Table:** 

GAPDH FW	AAGGTGAAGGTCGGAGTCAACGGATTTGGT
GAPDH REV	AGCCTTGACGGTGCCATGGAATTTGCCATG
CYCLIN E FW	CGTGCGTTTGCTTTTACAGA
CYCLIN E REV	AGCACCTTCCATAGCAGCAT
CCNB1 FW	CGGGAAGTCACTGGAAACAT
CCNB1 REV	AAACATGGCAGTGACACCAA
CDK2 FW	GCCCTAATCTCACCCTCTCC
CDK2 REV	AAGGGTGGTGGAGGCTAACT
FGF1 FW	GTGGATGGGACAAGGGACAG
FGF1 REV	ATTTGGTGTCTGTGAGCCGT
PAG1 FW	TCATTGCTGGGAAAAGGCCAAGA
PAG1 REV	AGAGCTGTGTCCTTGGAGGAA
SOCS3 FW	GGAGGTGACGAGCCCCC
SOCS3 REV	AAACTTGCTGTGGGTGACCA

### Statistical Analysis

Statistics was evaluated using GraphPad Prism 8 version (GraphPad Software). When two conditions were analyzed, Student’s unpaired *t*-test was used. When three or more conditions were analyzed, one-way ANOVA was used followed by Tukey’s post-test. For all in vitro experiments, data are reported as mean ± standard error mean (SEM). The level of statistical significance was set at *p*-value = 0.05.

## Results

### Characterization of BM-MSCs Expanded Inside the Nichoid

BM-MSC surface antigens were evaluated by flow cytometry to confirm mesenchymal features. CD90, CD73, CD105, and HLA-I surface antigens were expressed on more than 95% of MSCs, while less than 5% resulted positive for CD31, CD34, CD14, and CD45 molecules resulting compliant to the criteria defined by the International Society for Cellular Therapy [44].

**Fig. 1:**
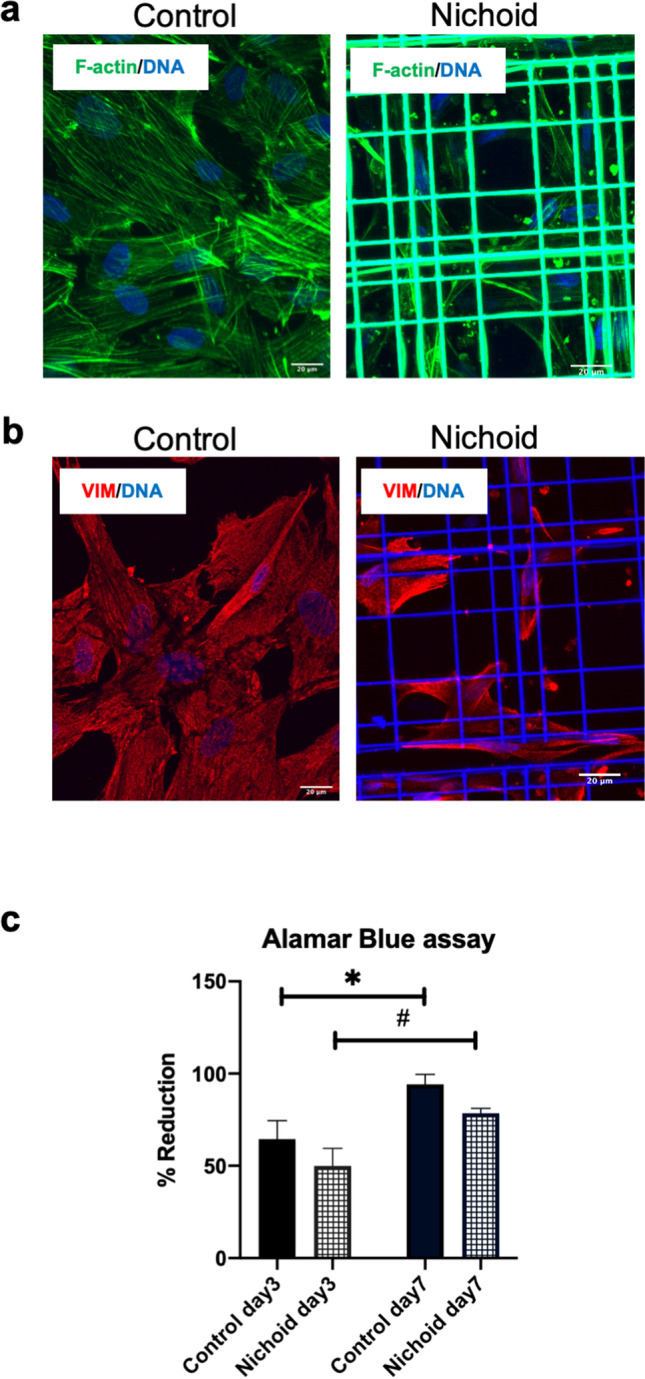
Characterization of BM-MSCs growth inside the Nichoid. **a** Immunofluorescence images of alpha-actin filaments in green (Phalloidin FITC) and nuclei in blue (Hoechst) in BM-MSCs expanded inside the Nichoid and 2D control. **b** Immunofluorescence analysis of mesenchymal cytoskeletal Vimentin (red) and nuclei in blue (Hoechst) in BM-MSCs expanded inside the Nichoid and control standard conditions. Scale bar: 20 μm. **c** Alamar Blue assay of BM-MSCs expanded inside the Nichoid and in control condition at day 3 and day 7 after seeding. Data are expressed as mean of 3 independent experiments ± SEM. *p < 0.05 control day 3 vs control day 7. #*p* < 0.05 Nichoid day 3 vs Nichoid day 7

BM-MSCs (10^4^ /1.9 cm^2^) were cultured inside the Nichoid scaffold and in standard conditions (used as control) for 7 days and then fixed for immunofluorescence analysis. In Fig. 1a, nuclei are stained in blue (Hoechst), and alpha-actin filaments are stained in green using Phalloidin-FITC, giving a qualitative information on the distribution of alpha-actin filaments. BM-MSCs grown on the flat substrate have a highly organized and large cytoskeleton, characterized by the presence of orientated filaments along the entire body of the cell. Nichoid-expanded BM-MSCs exhibit a different cytoskeletal organization: alpha-actin filaments are thin and form a more enhanced cortical actin network in comparison to cells in control conditions (Fig. 1a). These observations demonstrate that the geometrical and physical constrains of the Nichoid affect BM-MSCs morphology. In Fig. 1b, the positivity to Vimentin staining, a mesenchymal cytoskeletal marker, demonstrates that BM-MSCs maintain the mesenchymal feature when expanded inside the Nichoid, showing that the maintenance inside the 3D micro-scaffold does not determine spontaneous differentiation. In order to evaluate the viability of BM-MSCs grown inside the Nichoid, a metabolic Alamar Blue assay was performed. The Alamar Blue solution was incubated at 3 and 7 days after seeding (please see the “Materials and Methods” for details). As reported in Fig. 1c, there is a significative increase in metabolic activity from day 3 to day 7 with a comparable trend both in 3D and flat conditions. At day 3, the reduction levels of control cells and Nichoid-expanded cells were 64.49 ± 20.18% and 49.91 ± 18.96%, respectively. At day 7, the measured reduction value was 94.20 ± 13.30% for control cells and 78.51 ± 5.97% for Nichoid cells. The high metabolic activity levels observed in BM-MSCs grown inside the Nichoid for 7 days suggest that the structure does not alter the viability of the cells. The synthetic niche is thus suitable for the expansion of BM-MSCs.

**Fig. 2:**
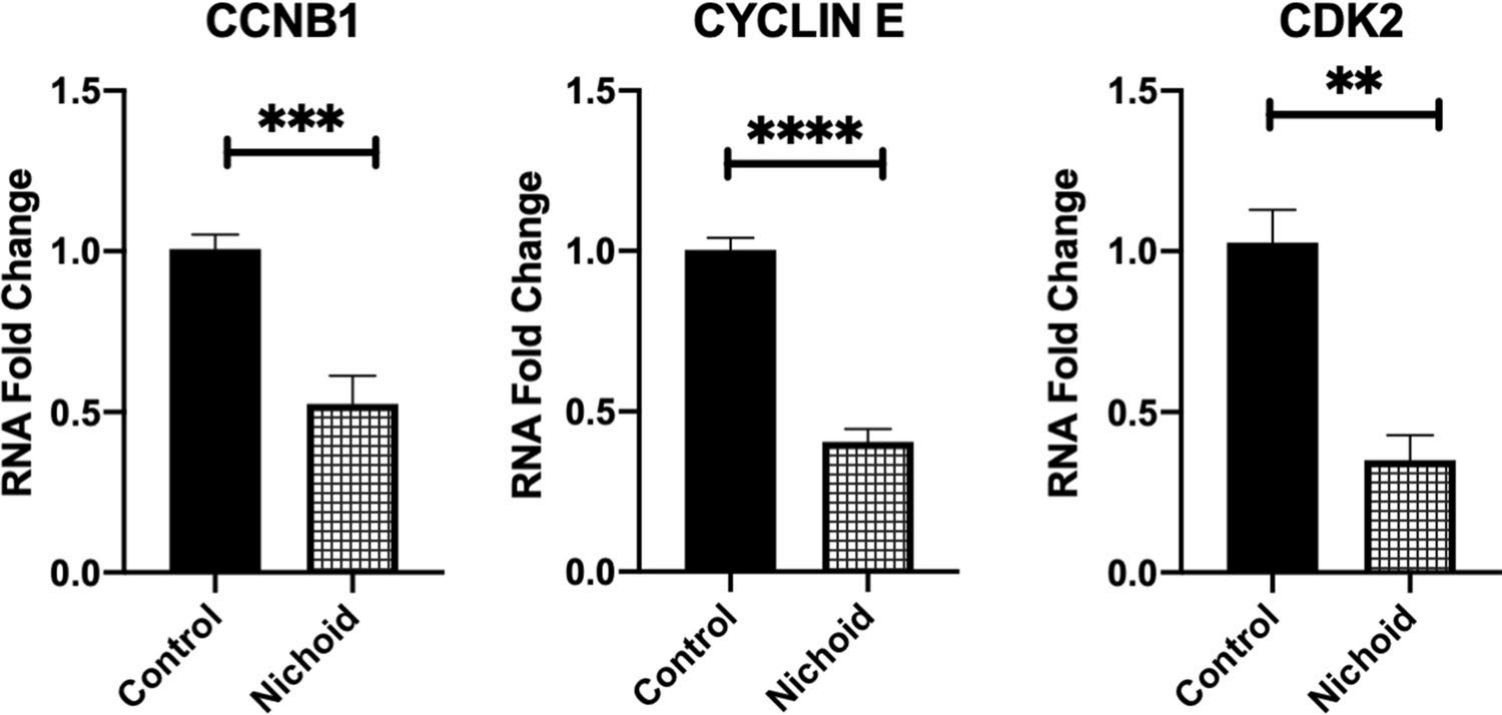
BM-MSCs expanded inside the Nichoid show the physiological quiescent state. Cyclins markers gene expression by real-time PCR of BM-MSCs expanded inside the Nichoid with respect to standard conditions. Data are expressed as the mean of three independent experiments with similar results ± SEM. **p < 0.01, ***p < 0.001, ****p < 0.0001 vs controls

Moreover, the expression of cell cycle markers was assessed by real-time PCR to evaluate how BM-MSCs’ proliferation differs inside the Nichoid with respect to the control condition. Specifically, we analyzed different checkpoints in the cell cycle such as cyclin B1 (CCNB1), a regulatory protein involved in the control of the cell cycle at the mitosis G2/M transition [45], cyclin E1 regulator of CDK kinases essential for G1/S transition during cells cycle [46], and cyclin-dependent kinase 2 (CDK2) serine/threonine protein kinase regulator of progression during the cell cycle [47]. In Fig. 2, a significative downregulation of all investigated cyclins was observed in BM-MSCs grown inside the Nichoid compared to the standard conditions, suggesting a quiescence state such as the one present in the physiological stem cell niche [48, 49].

### Gene Expression of the Nichoid-Expanded BM-MSCs

**Fig. 3:**
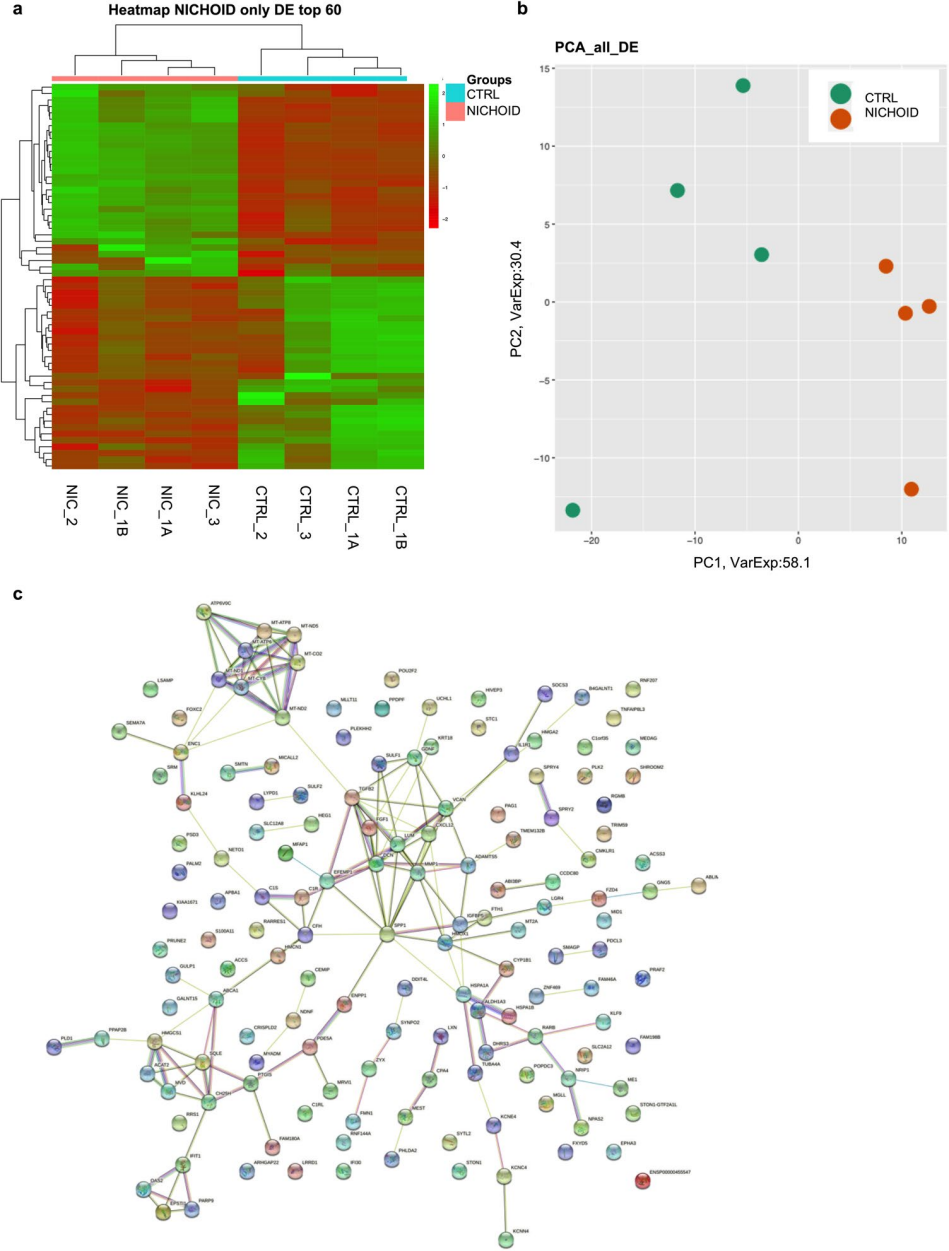
Transcriptome profile in BM-MSCs expanded inside the Nichoid vs standard conditions. **a** Expression profiles of differently expressed genes in Nichoid-grown BM-MSCs vs standard conditions reported as a heatmap. We considered as differentially expressed only genes showing |log2(Nichoid samples/Control samples)|≥ 1 and a False Discovery Rate ≤ 0.1; up-regulated genes are represented in green and down-regulated genes in red. **b** Expression profiles of significative differently expressed genes in Nichoid-grown BM-MSCs vs standard conditions reported as principal component analysis (PCA). **c** STRING interaction network based on significative deregulated genes where the nodes are proteins, and the edges represent the predicted functional connections. The combined score is computed by considering the probabilities from the different evidence channels corrected for the probability of randomly observing an interaction

Differential gene expression in BM-MSCs grown inside the Nichoid was evaluated with respect to controls. The gene expression profiles are reported in Fig. 3: heatmap (Fig. 3a) and PCA analyses (Fig. 3b) show different expression profiles between the two conditions, suggesting the influence of the Nichoid on specific cellular features. The heatmap displays the differentially expressed genes through a color code: up-regulated genes are represented in green and down-regulated genes in red. The clustering analysis reported on the top of the heatmap divided Nichoid samples and control samples in two different “families”: Nichoid samples were colored in pink, and control samples were colored in light blue. Indeed, Nichoid samples (*N* = 4) were grouped together and separated from control samples (*N* = 4). As expected, biological replicates within each group presented similar expression profiles, as shown by the color code. Similarly, PCA analysis of the DE RNAs in BM-MSCs expanded inside the Nichoid showed different expression profiles. Nichoid samples (orange) were grouped together and were well separated from control samples (green). Finally, all deregulated genes were subjected to a network STRING analysis to evaluate the links, and the most connected dysregulated gene is fibroblast growth factor 1 (FGF1) in Fig. 3c.

Moreover, we investigated the affected pathways related to the significantly dysregulated genes via the g:Profiler web tool. Specifically, we report the top 20 deregulated pathways found with the Reactome database, highlighting the pathways altered following BM-MSCs expansion inside the Nichoid. In Table 1, we reported the names of dysregulated pathways with the relative *p*-value, the total number of involved genes for the specific pathway, and the involved number of genes in our analysis. Finally, we also reported the name of specific genes that are dysregulated in the pathways. Interestingly, this analysis demonstrated an alteration in pathways related to cell metabolism and immune response.

**Table 1:** Reactome top 20 deregulated pathways performed by g:Profiler web tool. The table reports the name of deregulated pathways with the relative *p*-value, the total number of involved genes for the specific pathway, and the involved number of genes in our analysis. The fifth column depicts the name of the specific genes dysregulated in the pathways

Perturbated pathways	Adjusted *p*-value	Term size	Intersection size	Intersections
Metabolism	9,63835E-13	2075	31	GNG5, NPAS2,ACSS3,CEMIP,MT-ND2,PTGIS,PLD1,MVD,ABCA1,MT-CO2,MGLL,MT-ND1,HMGCS1,PARP9,MT-ATP8,MT-ND5,MT-ATP6,CH25H,VCAN,MT-CYB,HMOX1,B4GALNT1,SQLE,SRM,ACAT2,TNFAIP8L3,ME1,ENPP1,LUM,DCN,CYP1B1
Metabolism of lipids	1,82E-07	728	15	NPAS2, ACSS3,PTGIS,PLD1,MVD,ABCA1,MGLL,HMGCS1,CH25H,B4GALNT1,SQLE,ACAT2,TNFAIP8L3,ME1,CYP1B1
Respiratory electron transport, ATP synthesis by chemiosmotic coupling	4,50E-07	127	7	MT-ND2, MT-CO2,MT-ND1,MT-ATP8,MT-ND5,MT-ATP6,MT-CYB
The citric acid (TCA) cycle and respiratory electron transport	1,00074E-06	177	8	MT-ND2,MT-CO2,MT-ND1,MT-ATP8,MT-ND5,MT-ATP6,MT-CYB,ME1
Immune system	1,00074E-06	2041	19	HSPA1B,PAG1,C1S,MT2A,ATP6V0C,TUBA4A,PLD1,CRISPLD2,SOCS3,IFIT1,CFH,OAS2,HSPA1A,IL1R1,IFI30,FTH1,MMP1,HMOX1,C1ORF35
Signal transduction	3,79281E-06	2514	15	GNG5,PDE5A,PAG1,ARHGAP22,ALDH1A3,ATP6V0C,GDNF,TUBA4A,DHRS3,PLD1,FGF1,EPSTI1,SOCS3,ABCA1,MGLL
Extracellular matrix organization	6,11705E-06	298	9	EFEMP1,MFAP1,SPP1,VCAN,MMP1,LUM,ADAMTS5,TGFB2,DCN
Cholesterol biosynthesis	9,25359E-06	24	4	MVD,HMGCS1,SQLE,ACAT2
Metabolism of steroids	3,23404E-05	148	6	PTGIS,MVD,HMGCS1,CH25H,SQLE,ACAT2
Respiratory electron transport	5,7508E-05	103	5	MT-ND2,MT-CO2,MT-ND1,MT-ND5,MT-CYB
Interferon gamma signaling	7,76787E-05	87	5	MT2A,SOCS3,OAS2,IFI30,MID1
Cellular responses to stimuli	0,000101278	765	8	NPAS2,HSPA1B,MT2A,ATP6V0C,TUBA4A,MT-CO2,HMGA2,HSPA1A
Interferon Signaling	0,000207149	193	6	MT2A,SOCS3,IFIT1,OAS2,IFI30,MID1
Signaling by nuclear receptors	0,000207149	291	7	GNG5,ALDH1A3,DHRS3,ABCA1,NRIP1,RARB,CXCL12
Neuronal System	0,000277254	400	5	KCNC4,APBA1,GNG5,KCNN4,TUBA4A
Potassium Channels	0,000298821	103	3	KCNC4,GNG5,KCNN4
Cellular responses to stress	0,000669883	751	7	NPAS2,HSPA1B,ATP6V0C,TUBA4A,MT-CO2,HMGA2,HSPA1A
Disease	0,000780704	1639	15	GNG5,TUBA4A,FGF1,ABCA1,HSPA1A,PARP9,IL1R1,VCAN,PLK2,HMOX1,LUM,FZD4,ADAMTS5,DCN,CYP1B1
Neutrophil degranulation	0,000897936	476	7	HSPA1B,ATP6V0C,PLD1,CRISPLD2,HSPA1A,FTH1,C1ORF35
HSP90 chaperone cycle for steroid hormone receptors (SHR) in the presence of ligand	0,000897936	57	3	HSPA1B,TUBA4A,HSPA1A

Following the pathway analysis performed on the deregulated genes, to better investigate the effect of the Nichoid on BM-MSCs gene expression profile, we focused our attention on the top 10 deregulated coding genes for log_2_FC values (Table 2). These genes were involved in different pathways, such as cell adhesion and morphology, enzymatic activity, and immune response (as reported in the GeneCards database).

**Table 2:** Top 10 deregulated genes identified after transcriptome analysis. Gene function description was obtained from the GeneCards database (https://www.genecards.org/)

Gene	Log_2_FC	Description
EPHA3 EPH Receptor A3	2.28	It belongs to the ephrin receptor subfamily of the protein-tyrosine kinase family. The encoded protein is a receptor tyrosine kinase that leads to contact-dependent bidirectional signaling into neighboring cells. It is involved in the regulation of cell–cell adhesion, cytoskeletal organization, and cell migration
ACSS3 Acyl-CoA Synthetase Short Chain Family Member 3	2.14	It encodes a protein that catalyzes the synthesis of acetyl-CoA from short-chain fatty acids. It is a key enzyme for the activation of propionate metabolism, and it is required for acetate utilization and histone acetylation
PAG1 Phosphoprotein Membrane Anchor With Glycosphingolipid Microdomains 1	2.03	It encodes a type III transmembrane adaptor protein that binds to the tyrosine kinase csk protein. It is thought to be involved in the regulation of T-cell activation. It promotes cytoskeletal activation and recruitment to lipid rafts. It may be involved in cell adhesion signaling
RHGAP22 Rho GTPase Activating Protein 22	2.02	It encodes a member of the GTPase activating protein family which activates a GTPase belonging to the RAS superfamily of small GTP-binding proteins
RP11-20I23.1	2.02	It encodes a protein with a conserved domain, referred to as the TBC domain, characteristic of proteins which interact with GTPases. TBC domain proteins may serve as GTPase- activating proteins for a particular group of GTPases involved in the regulation of membrane trafficking
RARRES1 Retinoic Acid Receptor Responder 1	− 2.64	It is identified as a retinoid acid (RA) receptor-responsive gene. It encodes a type 1 membrane protein, which is an inhibitor of the cytoplasmic carboxypeptidase AGBL2. It may regulate the alpha-tubulin tyrosination cycle
APBA1 Amyloid Beta Precursor Protein Binding Family A Member 1	− 2.70	It encodes a member of the X11 protein family. It is a neuronal adapter protein that interacts with the Alzheimer's disease amyloid precursor protein (APP). It is also regarded as a putative vesicular trafficking protein in the brain that can form a complex with the potential to couple synaptic vesicle exocytosis to neuronal cell adhesion
MEST Mesoderm Specific Transcript	− 2.78	It encodes a member of the alpha/beta hydrolase superfamily. The encoded protein may play a role in development. It is a distinct gene associated with adipocyte differentiation and proliferation
KCNC4 Potassium Voltage-Gated Channel Subfamily C Member 4	− 2.92	It encodes components of voltage-gated potassium channels. This protein mediates the voltage-dependent potassium ion permeability of excitable membranes
LSAMP Limbic System Associated Membrane Protein	− 3.33	It encodes a member of the immunoglobulin LAMP, OBCAM and neurotrimin (IgLON) family of proteins. It mediates selective neuronal growth and axon targeting. It contributes to the guidance of developing axons and neural growth in the limbic system

**Fig. 4:**
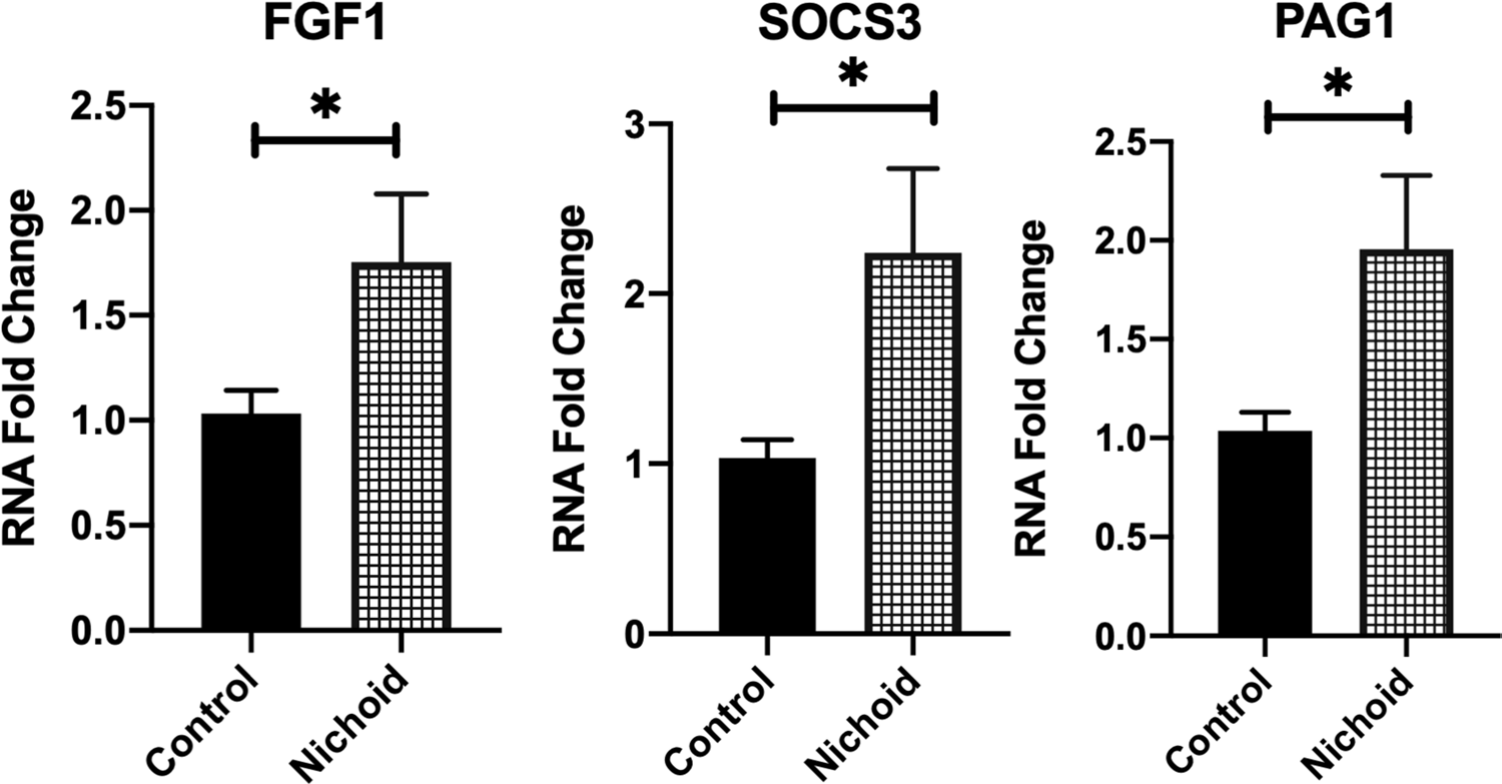
Validation of transcriptomic profile between BM-MSCs expanded inside the Nichoid vs standard conditions. Genes involved in immunomodulatory pathways were analyzed by real-time PCR in BM-MSCs expanded inside the Nichoid with respect to control standard conditions. Data are expressed as the mean of three independent experiments with similar results ± SEM. *p < 0.05 vs control

Finally, to validate RNA-seq analysis by real-time PCR, we selected three important genes implicated in the regulation of key cellular features. FGF1 resulted significatively up-regulated in BM-MSCs expanded inside the Nichoid with respect to controls (Fig. 4). This gene is involved in cell survival activities, such as cell growth, morphogenesis, tissue repair, tumor growth, and invasion. At the same time, two other genes implicated in the immune response and suppression of cytokine signaling were analyzed to validate RNA-seq analysis. PAG1, one of the most deregulated genes involved in T-cell activation and in immune system pathways, resulted up-regulated. Moreover, suppressor of cytokine signaling 3 (SOCS3), part of the previous presented IL-18 and interferon gamma pathways, resulted significantly up-regulated in BM-MSCs from Nichoid with respect to control conditions.

## Discussion and Conclusion

Numerous in vitro and in vivo studies demonstrated the beneficial effects of MSCs in the treatment of chronic, autoimmune, and auto-inflammatory diseases, including lung disorders [50]. In this work, the limits of 2D standard expansion were overcame exploiting the 3D scaffold Nichoid. BM-MSCs were fully characterized through several techniques, and it was demonstrated that the Nichoid mimics the physiological stem niche, maintaining the stem cell potential. Indeed, recent works demonstrate the stemness maintenance of neural stem cells of murine origin [35, 51, 52], but in this work, for the first time, we characterized human bone marrow mesenchymal stem cells growth inside the Nichoid with a specific focus on the anti-inflammatory gene expression profile.

BM-MSCs expanded inside the Nichoid created an actin cortex at the cell-grid interface and the consequent focal adhesions formation simulated an ex-vivo physiological niche, as previously demonstrated by Jacchetti et al. with rat mesenchymal stem cells [53]. Moreover, the Vimentin staining, a mesenchymal stem marker, was still present in BM-MSCs expanded inside the Nichoid, demonstrating that MSCs culture inside the Nichoid do not undergo spontaneous differentiation. The effects of the synthetic niche on the metabolic activity of BM-MSCs were studied, and a comparable increase in metabolic activity was observed in both culture conditions, suggesting that the synthetic niche is suitable for BM-MSCs culture. We highlighted a decreasing trend in the metabolic activity for BM-MSCs expanded inside the Nichoid with respect to the control condition. This is also concordant with results obtained on cyclins mRNA expression by real-time PCR analysis. Indeed, BM-MSCs expanded inside the Nichoid expressed significant lower levels of cell cycle positive regulators (cyclin E, CDK2, and CCNB1) with respect to the control condition. The downregulation of these genes indicated the cell cycle arrest at the G0 phase [54–56]. It has already been demonstrated that stem cells remain in a reversible quiescent state inside the stem niche, preserving the self-renewal potential [48, 49, 57, 58]. Indeed, BM-MSCs did not over-proliferate and remained in a quiescent state inside the synthetic niche, preserving stemness potential and preventing spontaneous differentiation, confirming the potential application of the Nichoid for stem cell expansion.

In order to acquire more in-depth insights over the effects of the Nichoid on cellular responses, a transcriptome analysis via RNA sequencing was performed. This technique allowed to observe significative differential gene profiles between BM-MSCs expanded in the micro-scaffold and the respective bidimensional controls. Deregulated genes were involved in several cellular pathways, including metabolism, morphology, enzymatic activity, and immune response, corroborating the hypothesis of a better response for cells expanded inside the Nichoid with respect to control condition. RNA sequencing revealed the Nichoid’s ability to impact on gene expression of BM-MSCs without the use of any chemical or xenogeneic factor, guaranteeing the safety of 3D scaffold-expanded cells.

A significative up-regulation of FGF1, SOCS3, and PAG1 in BM-MSCs expanded inside the Nichoid with respect to the control condition was observed. FGF1 is of critical importance in regulating stem cell pluripotency [59]. Indeed, FGF/FGFR signaling implicates fundamental cellular processes such as metabolism, cell survival, proliferation, migration, differentiation, embryonic development, and tissue regeneration. In 2017, Ghazavi et al. investigated the effect of intravenous administration of FGF1 transfected adipose-derived (AD)-MSCs for stroke therapy, observing a significant improvement of neurological functions and an increased density of FGF1 protein in the peri-infarct area [60]. At the same time, Liu et al. demonstrated that SCs could inhibit Th17 cell differentiation through the activation of SOCS3 suppressors, suggesting a possible mechanism through which MSCs could reduce pathological manifestations of some autoimmune diseases [61]. Finally, PAG1, which affected immune receptor signaling in T and B cells, was up-regulated and limited the inflammation effect. Indeed, Ullah et al. demonstrated that the PAG1 deficiency predisposed toward allergic sensitization and airways inflammation [62]. The results of gene up-regulation in BM-MSCs expanded inside the Nichoid, in agreement with the above-reported studies, suggest the improvement of the cells’ anti-inflammatory effects, possibly potentiating the therapeutic outcome of BM-MSCs. Although the injection of these targets has anti-inflammatory effects in several diseases and tumor suppression activity, possible side effects such as an over-proliferation stimulation could occurred [63]. However, a physiological production of these anti-inflammatory targets induced only by mechanical stimulation instead of a chemical and exogenous administration could reduce the possible side effects, potentially enhancing the stem cells’ safety. Moreover, preclinical and clinical results support the use of MSCs for therapeutic treatment also in lung diseases [9, 11], and in this work, the exploitation of the Nichoid could be a next ameliorating step for cell therapy production and hence clinical translation. Indeed, our results support the use of a 3D synthetic niche for the expansion of MSCs to render cellular and acellular products more suitable for clinical applications. These preclinical in vitro findings underline the possible clinical translation of the use of micro-scaffold as an MSCs culture expansion system for the future treatment of pediatric congenital and acquired pulmonary disorders, including post-COVID lung manifestations. To reach this important aim, more investigations, including in vivo experimental models, are needed.

## Data Availability

Data will be available upon reasonable request.
